# Efficacy of a compliant semicircular Ilizarov pin fixator module for treating infected nonunion of the femoral diaphysis

**DOI:** 10.1007/s11751-014-0199-6

**Published:** 2014-07-29

**Authors:** Ashraf A. Khanfour, Mohamed M. El-Sayed

**Affiliations:** 1 Department of Orthopaedic Surgery, Damanhur National Medical Institute, Damanhur, Egypt; 2 Department of Orthopaedic Surgery, Tanta University, Tanta, Egypt

**Keywords:** Femur, Ilizarov, External fixation, Infective nonunion, Compliant

## Abstract

Percutaneous transosseous Ilizarov wiring, whilst preferred in the tibia because of its unique properties, carries a high risk of complications in the femur. The aim of this work was to evaluate the efficacy of a more patient-friendly semicircular pin external fixator module built up from parts of the Ilizarov fixator components and its use in managing diaphyseal femoral nonunions. A group of 20 patients with infected diaphyseal nonunions of the femur after internal osteosynthesis were included in this study. The mean age of the patients at the time of surgery was 46 years (range 16–60, SD 15.6). The mean morbidity time since the original trauma was 10.2 months (range 6–15, SD 2.5). All the cases were fixed by the described external fixator module. Bony union with resolution of infection occurred in 18 (94.7 %) out of 19 cases after a mean period in the fixator of 11.2 months (range 8–18 SD 2.9). After a mean follow-up period of 3.5 years (range 2–9, SD 2.6), there were 14 excellent, 3 good, 1 fair and 1 poor results from radiological evaluation and 10 excellent, 7 good, 1 fair and 1 poor results from functional assessment. In conclusion, the described semi-circular pin fixator module is patient-friendly and effective in managing infected nonunions of the femoral diaphysis.

## Introduction

**Fig. 1:**
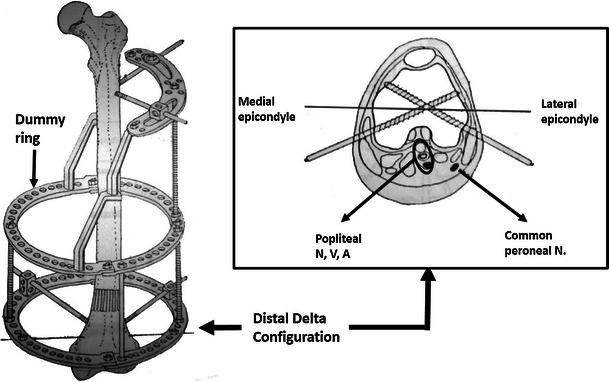
A hybrid Ilizarov external fixator of the femur with a dummy ring *(an empty ring without fixation to the bone. It is secured in the middle of the frame and acts as a force transmitter. It will effectively shorten the lengths of the rods and increases the stability of the frame)* and the distal delta configuration

A locked intramedullary nail is considered the method of choice for treating diaphyseal femoral fractures but is contraindicated in the presence of a concurrent or previous infection [1–5]. The percutaneously applied transosseous Ilizarov external fixator can be adopted for treating these cases, but there are disadvantages in patient compliance and interference with local anatomy [6]. These factors relate to a high risk of injury to the neurovascular structures during percutaneous transosseous wiring and tissue transfixation. Additionally, the application of the complete rings encircling the medial aspect of the patient’s thigh causes a major hindrance to a patient’s daily activities including personal hygiene [5, 7–13]. For these reasons, a classic Ilizarov fixator using tension wires in the proximal femoral segment is not popular [12, 14] leading to, in 1986, Italian surgeons Catagni and Cattaneo introducing the hybrid modification of using half-pins but maintaining all wires in the distal femur [11, 12]. This assembly has undergone many modifications including the introduction of concepts of a “dummy ring” and a more stable “delta” distal Schanz pin configuration (Fig. 1). Despite this, patient acceptance remains a major drawback [10, 12, 15–17].

This work evaluates the efficacy of a more patient-acceptable semicircular external fixator module built up from parts of the Ilizarov fixator components for managing diaphyseal femoral fractures.

## Patients and methods

A consecutive cohort of 20 patients with infected diaphyseal nonunions of the femur (after previously unsuccessful internal osteosynthesis) was treated using the semicircular fixator. The patients presented between January 2003 and February 2010. The study was approved by the local ethical committee (General Organization of Teaching Hospitals and Institutes Research Ethical Committee) and was conducted in accordance with the Declaration of Helsinki and Ethical Guidelines for Epidemiological Research (2008). Informed written consent was obtained from all patients and their guardians before participation in the study.

The presence of a nonunion was established clinically: the ability to induce motion at the fracture site; deep tenderness; deformity; and inability to bear weight. Using radiographs, the diagnosis was supported by the presence of a radiolucent gap at the fracture site, sealing off of the medullary cavity, sclerosis of the fractured bone edges or absence of bridging callus after 6 months of the fracture event with evidence of loosening of the implant. The diagnosis was made also if the fracture showed no progressive radiological signs of healing on three successive months.

Infection was suspected clinically by the presence of local pain out of proportion to the nonunion, erythema, swelling and induration with or without chronic draining sinuses. Further evidence was obtained through investigations, which added to a high probability of sepsis: a raised CBC, ESR; the presence of sequestrum, involucrum, periosteal and endosteal new bone formation, cortical irregularities and visible resorption, especially around the osteosynthesis implant on X-rays.

The inclusion criteria were cases with an established infected nonunion of the femoral diaphysis after internal fixation. The exclusion criteria were very obese patients with a large thigh diameter rendering the half-pin offset to be unacceptably long, an insensate limb and noncompliant patients.

### The construct (Fig. 2)

**Fig. 2:**
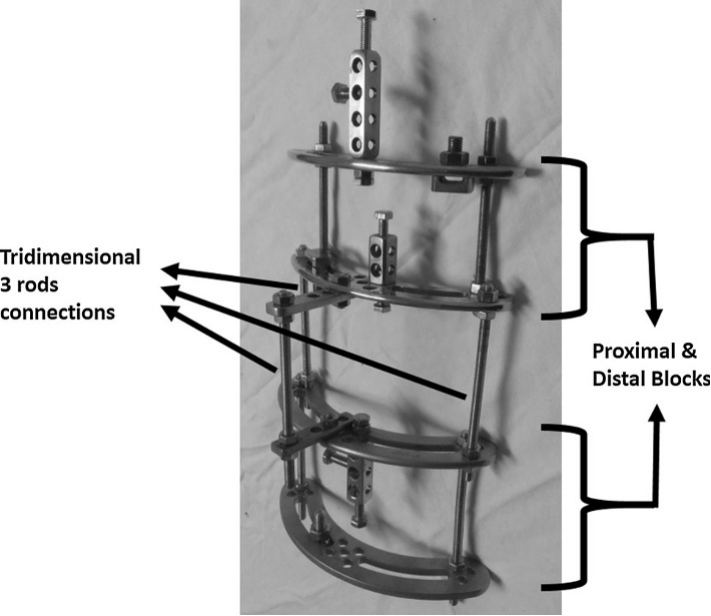
The semicircular femoral Ilizarov pin fixator module used

The frame was composed of two blocks. Each block comprised two identical femoral arches connected as a pair and to which pin clamps or Rancho cubes were fixed as required. The two blocks were connected by three threaded rods in which the middle rod was lateralised by the aid of two straight plates connected orthogonally to the middle of these arches to add to the multiplanar stability of the frame (Fig. 2). The length of the entire fixator covered the whole length of the femur. Six millimetre diameter Schanz half-pins were used mainly with the smaller 5 mm pins for smaller diameter femurs.

### Operative technique

**Fig. 3:**
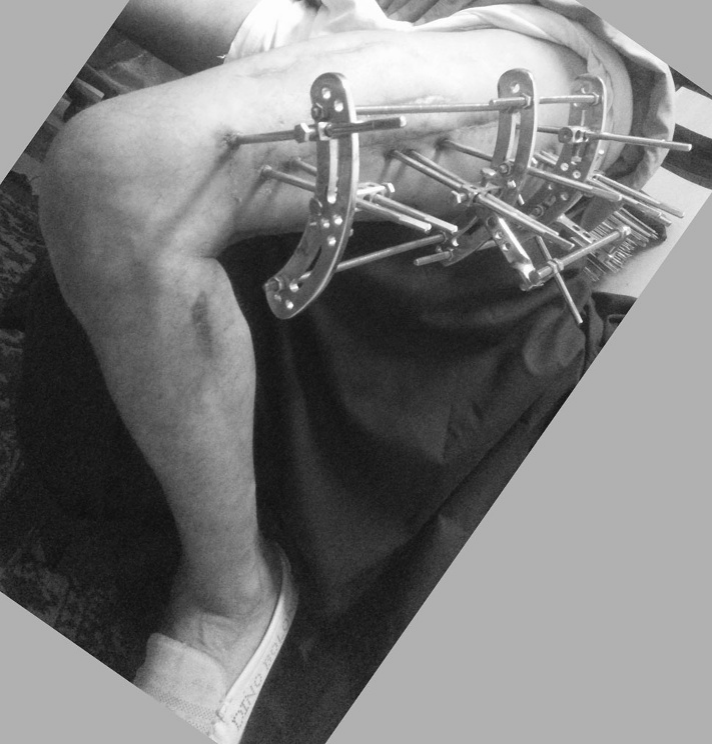
This photograph shows the divergence and the spread of the pins across the femur. It also demonstrates the ability of the patient to bend his knee freely beyond 90^°^

**Fig. 4:**
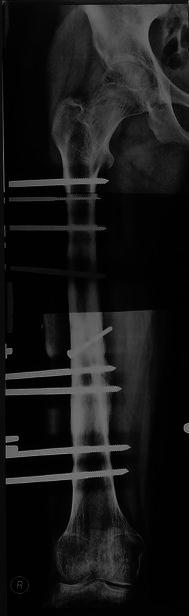
Case no. 1 in table I. A-P view X-ray showing infected nonunion with a spiral fracture configuration at the mid shaft right femur fixed by the described semicircular Ilizarov pin fixator module augmented by an interfragmentary cortical screw

**Fig. 5:**
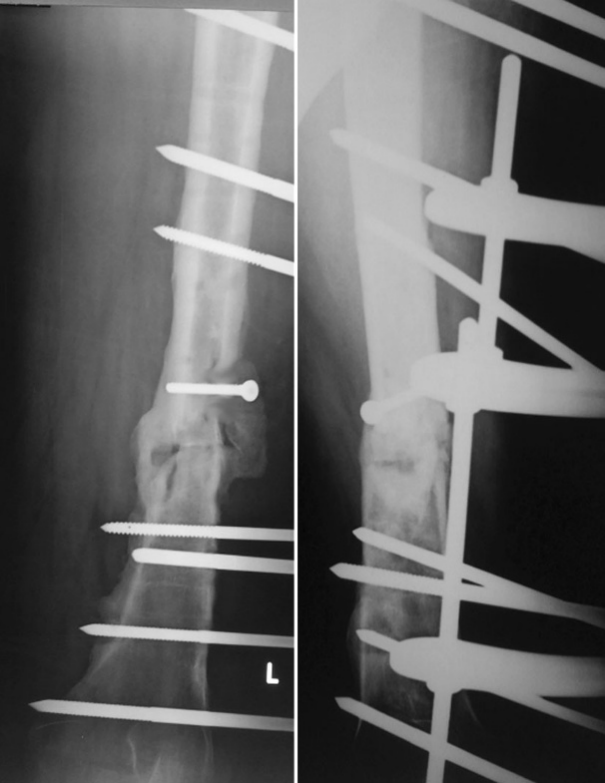
Case no. 8 in table I. Illustrates an augmentative structural iliac bone graft that was securely fixed by a cortical screw. Union of the femoral fracture was achieved after incorporation of the strut graft

The construct was assembled pre-operatively. A radiolucent table was used, and a pillow placed to support the ipsilateral buttock of the patient in the supine position. A first surgical strategy was to deal with the infected nonunion: a lateral approach, taking deep samples for culture and sensitivity, extraction of the implants and aggressive debridement of all devitalised soft tissue and bone. The bone ends were cut back square until punctate bleeding was clearly evident, and this followed by lavage with normal saline. The nonunion site was prepared for bone grafting by fish scaling (cortical flaps) and multiple drill holes. If the knee was mobile, the fixator was applied with the knee flexed to 90^°^ at the end of a radiolucent operating table to ensure the quadriceps were transfixed in flexion by the pins. The fixator was applied orthogonally to the femur by first inserting a 6 mm diameter Schanz screw (5 mm in small diameter femurs) reference pin at the subtrochanteric level near the lesser trochanter, perpendicular to the bone and in the coronal plane. The pre-assembled proximal block was fixed to this reference pin, and further pins added to hold the proximal segment securely. With an assistant holding the reduction of the newly prepared bone ends, fixation of the distal fragment to the fixator was then carried out. At the completion of fixation, each segment had at least three to four pins in different planes with as much of a divergence angle in between as possible (Fig. 3). The fixation pins were at least 3 cm from the fracture site. Augmentative interfragmentary screw fixation was needed for one case (Fig. 4). Structural iliac bone grafts were used in seven cases that were fixed with a screw (Fig. 5). Iliac bone chips were used in two cases to stimulate healing. Two cases needed repeat bone grafting, one after 9 months and the other after 10 months to stimulate healing. In none of the cases was simultaneous bone lengthening performed.

The patient was discharged on the second or third postoperative day. Early assisted weight-bearing and continual knee exercises were encouraged. Antibiotics were prescribed according to culture and sensitivity results from deep samples submitted and were for 2 weeks after resolution of clinical signs of infection. Follow-up X-rays were obtained every month for the first 3 months, thereafter every 2 months until union and removal of the fixator. Frequent compression of the fracture site was needed if osteolysis at the fracture site was noted. Union was declared through observing bridging callus on anteroposterior, lateral and oblique views; this was confirmed clinically by dynamising the fixator and observing the ability of the patient to walk and perform a single-leg stance on the affected limb without pain, instability or deformation at the fracture site. The fixator was removed in the operating room under general anaesthesia.

Outcome was determined using a combination of radiological and functional criteria. The classification system by Paley and Maar [18, 19] was used. This is based on the presence or absence of each of these five criteria: union; infection; deformity in any plane >5^°^; limb length discrepancy >2.5 cm; and a weak cross-sectional area at the union site that requires long-term bracing or protection. An *excellent* bone result was assigned for those who achieved full union and resolution of infection with absence of the other three factors. A *good* bone result was as excellent with the presence of one of the other three criteria. A *fair* bone result was as excellent with the presence of two of the other criteria. A *poor* bone result was a persistent nonunion with or without persistent or recurrent bone infection.

Additional outcome reporting used a modification of the system derived from Paley and Maar in 2000 and Barbarossa V et al. in 2001 [19, 20]. This was based on five criteria: the ability for normal daily activities and a return to work; pain; the need for walking aids or braces; a loss of more than 20^°^ of knee range of motion from the pre-operative levels; and soft tissue dystrophy. An *excellent* result was assigned for those with full activity, and the other four criteria were absent. A *good* result was deemed if the patient was active with mild or no pain, and one of the other three criteria present. A *fair* result was declared if the patient was active with mild or no pain, and two of the other criteria present. A *poor* occurred if the patient had markedly limited activity regardless of the presence of other criteria, any patient with significant pain (requiring narcotics) or a patient with three of the other criteria.

## Results

The mean age of patients at the time of surgery was 46 years (range 16–60, SD 15.6), 16 (80 %) were male and 4 (20 %) were females. The mean period since the original trauma was 10.2 months (range 6–15, SD 2.5). A history of road traffic accident was noted in 18 patients; one patient fractured after a fall (case no. 10), and one had a gunshot injury (case no. 7). Eighteen were initially closed fractures, and one was an open fracture grade II (Gustilo and Anderson Classification). The mean number of previous operative procedures was 3; these included repeated debridement, plating, intramedullary nailing, gentamicin bead implantation and external fixation. Four patients were diabetic and 1 positive for hepatitis C. One patient had ipsilateral anterior poliomyelitis of the lower limb.

Eleven patients presented with a stiff knee in extension. Eighteen had minor shortening <2 cm, whilst two patients presented with 3 and 2.5 cm shortening (cases no. 4 and 9), respectively. Five cases presented with >1/3 circumferential cortical defect (cases no. 2, 7–9, 13, 15, 18; Table 1).

**Table 1:** Patient demography

No.	Age (years)	Sex	Side	Presenting condition	General Comorbidity	Local Co-morbidity	No. of pre-op interferences	Period of nonunion (months)	Adjunctive synchronous procedure done	Further interference needed	Time spent in Ilizarov (M)	Union	Follow-up (years)
1	16	M	Rt	Infected non union with plate fixation	–	Stiff knee in extension	plating	7	Augmentative interfragmental screw	–	9	+	2
2	17	M	Rt	Infected non union with plate fixation	–	Stiff knee in extension	Plating. 2 time depridement	8	Strut Iliac bone graft	Re-grafting after 9 m	18	+	2
3	42	M	Rt	Infected non union with plate fixation	–	Stiff knee in extension	Plating. 3 time depridement	14	–	–	10	+	4
4	58	M	Rt	Infected non union with ILN fixation	Diabetic	Stiff knee in extension	ILN 10 time depridement. 1 time septobal beeds insertion.	12	–	–	9	+	2
5	58	M	Lt	Infected non union with plate fixation	–	–	plating	10	–	–	10	+	9
6	60	F	Lt	Infected non union with ILN fixation)	–	Stiff knee in extension	Plating. 3 time depridement	8	Iliac bone graft	–	9	+	3
7	46	M	Rt	Infected nonunion with Ilizarov hybrid fixation with intramedullary Ruch pin	–	Stiff knee in extension	Interlocking nail, that was extracted after 6 m	13	Strut Iliac bone graft	–	11	+	3
8	55	F	Lt	Infected nonunion with plating	–	–	Plating. 2 time depridement	9	Strut Iliac bone graft that was fixed by a screw	–	9	+	4
9	18	M	Rt	Infected nonunion with IMN	–	Stiff knee in extension	IMN. Orthofix for 3 m	13	Strut Iliac bone graft	–	9	+	2
10	58	M	Lt	Infected nonunion with a long DHS	Diabetic	–	DHS. 2 times depridement.	10	–	–	8	+	7
11	59	M	Lt	Infected nonunion with Orthofix fixation	Diabetic	Stiff knee in extension	Orthofix	10	–	–	13	+	4
12	59	M	Rt	Infected nonunion with plating	Diabetic	–	Plating	15	–	–	??	Missed	–
13	48	M	Rt	Infected nonunion with plating	Ipsilateral poliomyelitis	Stiff knee in extension	2times plating	8	Strut Iliac bone graft	–	11	+	3
14	58	M	Lt	Infected nonunion with plating	–	–	plating	6	–	–	11	+	9
15	55	F	Rt	Infected nonunion with plating	–	–	Plating. 4 time depridement	8	Strut Iliac bone graft	–	10	+	5
16	55	M	Rt	Infected nonunion with plating	Hepatitis C positive	Stiff knee in extension	Plate femur 4 time depridement	9	–	–	9	Not united	–
17	45	M	Lt	Infected nonunion with ILN	–	–	Interlocking nail. 4 time depridement	11	Iliac graft bone chips	–	13	+	6
18	53	M	Lt	Infected nonunion with ILN	–	–	ILN 3 time depridement.	9	Strut Iliac bone graft -	–	14	+	2
19	39	M	Rt	Infected nonunion with plating	–	–	Plating.	13	–	Re-grafting after 10 m	17	+	3
20	21	F	Lt	Infected nonunion with ILN	–	Stiff knee in extension	ILN	11	–	–	11	+	4

**Fig. 6:**
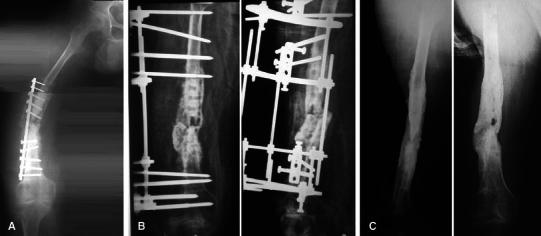
**a**–**c** Case no. 13 in table I. **a** A-P view of showing infected nonunion of the midshaft femur with periosteal and endosteal new bone formation, cortical irregularities and visible resorption, especially around the plate and screws with loosening of the fixation. **b** Postoperative A-P and lateral X-rays with the fixator. **c** Follow-up X-rays after 3 years. This was classed an excellent result from both bony and functional outcomes

The mean period spent in the fixator was 11 months (range 8–18, SD 2.9). After a mean follow-up period of 3.5 years (range 2–9, SD 2.6), bony union without recurrence of infection was noted in 18 out of 19 patients (94.7 %) who attended the final follow-up (Fig. 6). The patient lost to follow-up (case no. 12 in the table) was a 59-year-old diabetic male with an infected midshaft nonunion of the femur with two previous episodes of plate fixation. He was treated in the fixator for 8 months and then lost to follow-up.

Using the bony criteria, there were 14 excellent, 3 good, 1 fair and 1 poor result. Despite the inevitable shortening of the limb from freshening and squaring off the nonunion bone ends, only two patients showed significant shortening >2.5 cm (cases no. 4 and 9 with 5 and 4 cm, respectively) but were also the cases who presented initially with 3 and 2.5 cm shortening. The poor result (case no. 16 in the table) was a 55-year-old male with infected nonunion fracture of the upper to mid third right femoral shaft after previous plating. He was positive for hepatitis C infection with a stiff knee in extension. After 9 months in the fixator, he refused to continue with treatment and asked for removal of the fixator and substitution with an ischial weight-bearing orthosis. Using functional criteria, there were 10 excellent, 7 good, 1 fair and 1 poor result (Table 2).

**Table 2:** Bone and functional results

No.	Bone results	Functional results
1	G (Frontal plane varus 8^°^)	E
2	F (Weak union site cross-sectional area that necessitates long bracing for 9 months)	F (Soft tissue dystrophy and need walking brace for 9 months)
3	E	G (Soft tissue dystrophy)
4	G (LLD 5 cm)	E
5	E	E
6	E	G (Soft tissue dystrophy)
7	E	E
8	E	E
9	G (LLD 4 cm)	G (Need walking brace for 10 months)
10	E	E
11	E	G (Loss of >20^°^ knee ROM from the presenting range)
12	Missed case	
13	E	E
14	E	E
15	E	G (Loss of >20^°^ knee ROM from the presenting range)
16	P (Nonunion)	P (Marked limitation of daily activity)
17	E	E
18	E	G (Need for walking aid)
19	E	E
20	E	G (Loss of >20^°^ knee ROM from the presenting range)

*E* excellent result, *G* good result, *F* fair result, *P* poor result

During the period of external fixation, patients noted easier compliance with this fixator in carrying out their physiological daily activities, personal hygiene, sitting or lying down. All patients had one or more pin track infections that were resolved by local dressing and systemic antibiotics. There were bouts of pain, oedema and reactive depression, all of which resolved after completion of treatment. A refracture occurred in one patient 2 years after completion of treatment following new trauma and was managed by the same fixator; this united after 8 months. Of the eleven patients who presented with stiff knees in extension, none showed any noticeable improvement at the end of follow-up. For those patients who developed loss of knee motion during the period of fixation, this was noted to improve by the end of follow-up with the exception of three cases (Table 2).

## Discussion

When external fixation is indicated, a balance between the desirable characteristics of the fixator assembly and patient tolerability is important [7, 21]. Biomechanically and in the femur, unilateral fixators are weaker in bending, axial and torsional stability. In contrast, ring fixators give excellent three-dimensional stability but at the expense of the patient comfort. Even in a hybrid fixator, the distal transfixing wires (apart from being difficult to tolerate) are not sufficiently rigid against bending forces in the sagittal plane as the permitted safe corridor for insertion is narrow and located mainly in the frontal plane [7, 22].

Factors which determine the stiffness of a fixator construct depend on its two main constituents: the device material and configuration on one hand and the pins on the other. Fixator rigidity is directly proportion to pin stiffness that is increased by: firstly improving material properties; secondly by increasing pin diameter—but not to exceed one-third of the bone diameter—and thirdly a decreased pin offset (the free bending length of pins which is the distance between the bone surface and the external pin clamp). Other pin factors that add to fixator rigidity are increased pin spread (length of the fractured bone involved in fixation), increased pin numbers and levels and lastly, increased pin divergent angle in the axial plane [7, 11, 21–25]. Considering the aforementioned factors, the design of the external fixator used in this study can be considered as a modification of the Catagni and Cattaneo module in 1986 [11, 12] but where the distal rings are replaced by femoral arches.

From a patient’s perspective of fixator tolerability and comfort, this fixator conveniently spares encircling the medial aspect of the patient’s thigh with bulky rings. From a biomechanical point of view, it is a versatile semicircular multiplanar device. It allows for frequent compression of the fracture site postoperatively when fracture site resorption is observed. The mechanical axis of the femur lies outside the bone itself and medial to its anatomical axis. Axial loading on the femoral head will create compression forces on its medial side and a tensile force on the lateral side. Consequently, stable fixation of a femoral diaphyseal fracture is accomplished if, provided that inherent bone stability (through fracture site bone contact) was obtained first, a sufficiently rigid external fixator is applied laterally and is sufficient to act as a tension band fixation [5, 10, 26–28].

Inherent bone stability and bone contact, especially of the medial cortex, are of utmost importance for fixation stability that enables the patient to weight bear early. Bone contact increases the effective diameter of the fracture to obtain a good cross-sectional area of bone at the future union site [5, 22, 29]. This was achieved using different ways of squaring off the bone ends and occasional use of supplemental minimal osteosynthesis (interfragmentary screws) to stabilise unstable oblique fractures or with structural grafts to fill a partial circumferential bone defect if this was >1/3 diameter of the femur.

Concomitant bone lengthening for cases with infected nonunion of the femur has been reported to carry significant complications [20, 30]. These findings were also reported again by Blum et al. in 2010 [30]. This suggests deferring lengthening—if required—to a second stage after achieving full fracture consolidation and recovery of the patient’s physical, functional and psychological status. It should be reserved for those who are able to cope with the strenuous combined treatment protocols of external fixation and lengthening. Fortunately, it has been reported that patients may tolerate shortening well up to 2 cm without the need for a shoe lift and, for those with up to 4 cm shortening, comply well with a shoe lift of 2 cm [31, 32].

The final results presented in this consecutive series are comparable with those from similar studies [2–4, 20, 33]. The mean time in the fixator was 11.2 months and is comparable to other published works [2–4, 20]. The prevalence of knee stiffness is not always a complication of the use external fixation but is related to other factors such as the fracture location, the extent of soft tissue damage, pre-exiting stiffness and the severity and duration of infection [3]. The limitations of this study include the absence of a control group and the small cohort of patients but this attributable to the restriction in the inclusion criteria to preserve group homogeneity.

## Conclusion

A semi-circular external fixator module which is entirely half-pin based is described, which is shown to be patient tolerable and effective for managing infected nonunion of the femoral diaphysis.
